# Genome-Based Genotype × Environment Prediction Enhances Potato (*Solanum tuberosum* L.) Improvement Using Pseudo-Diploid and Polysomic Tetraploid Modeling

**DOI:** 10.3389/fpls.2022.785196

**Published:** 2022-02-07

**Authors:** Rodomiro Ortiz, José Crossa, Fredrik Reslow, Paulino Perez-Rodriguez, Jaime Cuevas

**Affiliations:** ^1^Department of Plant Breeding, Swedish University of Agricultural Sciences (SLU), Lomma, Sweden; ^2^International Maize and Wheat Improvement Center (CIMMYT), Texcoco, Mexico; ^3^Colegio de Postgraduados, Montecillos, Mexico; ^4^División de Ciencias, Ingeniería y Tecnologías, Universidad de Quintana Roo, Chetumal, Mexico

**Keywords:** genomic-enabled predictions, multi-environment trials, potato breeding, *Solanum tuberosum*, genetic gains in plant breeding

## Abstract

Potato breeding must improve its efficiency by increasing the reliability of selection as well as identifying a promising germplasm for crossing. This study shows the prediction accuracy of genomic-estimated breeding values for several potato (*Solanum tuberosum* L.) breeding clones and the released cultivars that were evaluated at three locations in northern and southern Sweden for various traits. Three dosages of marker alleles [pseudo-diploid (A), additive tetrasomic polyploidy (B), and additive-non-additive tetrasomic polyploidy (C)] were considered in the genome-based prediction models, for single environments and multiple environments (accounting for the genotype-by-environment interaction or G × E), and for comparing two kernels, the conventional linear, Genomic Best Linear Unbiased Prediction (GBLUP) (GB), and the non-linear Gaussian kernel (GK), when used with the single-kernel genetic matrices of A, B, C, or when employing two-kernel genetic matrices in the model using the kernels from B and C for a single environment (models 1 and 2, respectively), and for multi-environments (models 3 and 4, respectively). Concerning the single site analyses, the trait with the highest prediction accuracy for all sites under A, B, C for model 1, model 2, and for GB and GK methods was tuber starch percentage. Another trait with relatively high prediction accuracy was the total tuber weight. Results show an increase in prediction accuracy of model 2 over model 1. Non-linear Gaussian kernel (GK) did not show any clear advantage over the linear kernel GBLUP (GB). Results from the multi-environments had prediction accuracy estimates (models 3 and 4) higher than those obtained from the single-environment analyses. Model 4 with GB was the best method in combination with the marker structure B for predicting most of the tuber traits. Most of the traits gave relatively high prediction accuracy under this combination of marker structure (A, B, C, and B-C), and methods GB and GK combined with the multi-environment with G × E model.

## Introduction

Potato (*Solanum tuberosum* L.) ranks among the most important crops in human diets worldwide after rice and wheat. The most widely grown potatoes are self-compatible polysomic tetraploid species (2*n* = 4*x* = 48), which show tetrasomic inheritance and inbreeding depression after continuous self-fertilizing. Potato is a vegetatively propagating crop in which each tuber is identical to its mother plant, thus, allowing favorable traits to be fixed in the F_1_ hybrid generation. Potato cultivars or breeding clones are often highly heterozygous, and tuber yield benefits from heterosis, which is a very important target in potato breeding. One of the major concerns is, however, stagnated tuber yield gains in potato cultivation (Douches et al., 1996; Guo, 2021). Tuber yield is a complex quantitative trait due to its multi-genic nature (Bradshaw, 2021), thus, making it difficult to evaluate in the early stages of the potato breeding cycle (Brown et al., 1987). Genome-based prediction (GP) based on genotyping, along with genome-wide single nucleotide polymorphisms, pedigree, and phenotypic data, is a very powerful tool to capture small genetic effects dispersed over the genome, which allows predicting an individual’s breeding value (Desta and Ortiz, 2014).

New methods and tools are continuously being developed to integrate GP in genetics research and to use them for breeding crops, livestock, and trees. Several genome-based models are being developed, including the family of additive Bayesian linear regression models, initially proposed by Meuwissen et al. (2001), and named the Bayesian alphabet. Mixed linear models with fixed effects described by the general mean (or intercept) or any other fixed effect and random genetic effects assuming a multivariate normal distribution with mean zero and covariance matrix $\sigma_{u}^{2}\,\text{K},$ where $\sigma_{u}^{2}$ is a scaled parameter reflecting the variance of random effects to be estimated, and **K** is a known matrix that expresses the genetic similarity of the individuals.

The most common genetic similarity covariance matrix between individuals used in genome-based prediction is the linear similarity kernel relationship matrix called genomic best linear unbiased prediction (GBLUP) (VanRaden, 2007, 2008). However, departures from linearity are usually the rule because complex cryptic interactions among genes (i.e., epistasis) and their interaction with the environment are part of the genetic composition of complex traits. These deviations from linearity are addressed by semi-parametric approaches, such as the non-linear Gaussian kernel (GK) of the reproducing kernel Hilbert space (RKHS) regression (Gianola et al., 2006, 2014; Cuevas et al., 2016, 2017). The RKHS regression reduces the dimension of the parametric space and captures small complex interaction among markers. Another non-linear kernel is the arc-cosine kernel (Cuevas et al., 2019) that attempts to emulate neural networks with multiple layers. Morota and Gianola (2014) mentioned that genome prediction coupled with combinations of kernels may capture a non-additive variation (Gianola et al., 2014).

In plant breeding, genotype × environment interaction (G × E) plays an important confounding role when selecting candidates to recombine. A common way to assess the extent of G × E in plant breeding and agricultural experiments is to estimate genetic correlations of performance across environments because these correlations summarize the joint action of genes and environmental conditions. The model proposed by Burgueño et al. (2012) represents the random genetic × environment effects modeled with a multivariate normal distribution with zero mean and the variance-covariance described as the Kronecker product between the genetic correlation between the **K** cultivars and the matrix of the relationship between **E**environments, constructed with environmental covariables or with an incident matrix of zeros. The model of Burgueño et al. (2012) has the advantage that it estimates the genetic covariance between environments.

Jarquín et al. (2014) proposed a class of random effects model where the main effects of genomic and environmental covariates (Ecs), as well as the interactions between them, are introduced using covariance structures that are the functions of marker genotypes and Ecs. The proposed approach represents an extension of the GBLUP and can be interpreted as a random effects model on all the markers, all the Ecs, and all the interactions between markers and Ecs using a multiplicative operator. Jarquín et al. (2014) proposes modeling the variance-covariance G × E by the Hadamard product between the random genetic effects and the random environmental effects. The main advantage of this model is that it allows using environmental climatic covariables that are measured in each environment during the cropping season. In general, a multi-environment model –including modeling the G × E as described above– improved the genome-based prediction accuracy (Burgueño et al., 2012; Jarquín et al., 2014; Cuevas et al., 2016, 2017, 2018; Sousa et al., 2017; Granato et al., 2018). Recently, Martini et al. (2020) explained the relationship between Kronecker and Hadamard products for modeling G × E.

Lopez-Cruz et al. (2015) proposed a marker × environment interaction model, where the marker effect and genomic values are partitioned into components that are stable across environments (main effects), and others that are environment-specific (interactions). This interaction model is useful when selecting for stability and for adaptation to targeted environments. This marker × environment interaction model is easy to implement in standard software for genomic selection (GS), and it can also be implemented with any priors commonly used in GS, including not only the shrinkage methods (e.g., GBLUP), but also the variable selection methods that could not be directly implemented under the reaction norm model, as indicated by Crossa et al. (2016). The marker × environment interaction model of Lopez-Cruz et al. (2015) is appropriate for sets of environments that are positively correlated. However, in practice, this G × E pattern may be too restrictive in cases where several environments have zero or negative correlation with each other or with others.

Cuevas et al. (2016) applied the marker × environment interaction GS model of Lopez-Cruz et al. (2015) after modeling through the standard linear kernel (GBLUP), as well as by a non-linear Gaussian kernel similar to that used in the RKHS with kernel averaging (RKHS KA) (de los Campos et al., 2010), and the Gaussian kernel with the bandwidth estimated through an empirical Bayesian method (Pérez-Elizalde et al., 2015). The methods proposed by Cuevas et al. (2016) were used to perform single-environment analyses and extended to account for G × E interaction in wheat and maize datasets. For single-environment and multi-environment analyses, the Gaussian kernel showed accuracies up to 17% higher than that of the multi environment G × E interaction model with GBLUP. Cuevas et al. (2016) concluded that the higher prediction accuracy of the Gaussian kernel models, coupled with the G × E model, is due to more flexible kernels that allow the accounting for small, more complex marker main effects, and marker-specific interaction effects.

Genomic prediction models were initially sought for predicting tuber yield in potato with a prediction accuracy between 0.2 and 0.4, but a model including additive and dominance effects may increase it (Ortiz, 2020, and references therein). Although genomic prediction of breeding values seems to be feasible in potato, these predictions across breeding populations remain in their infancy owing to the high allelic diversity in this crop, which calls for carefully defining the training sets. Furthermore, the genome-based prediction of the tetrasomic potato includes the complexity of having to determine the dosage of the different marker alleles for the possible genotypes AAAA, AAAB, AABB, ABBB, and BBBB. Slater et al. (2016) considered the marker dosage and computed the expected accuracy of genomic selection for traits with different heritability, and compared the genetic gains from genomic selection with those from phenotypic selection. The authors found that genomic selection can increase genetic gains in tetrasomic cultivated potato.

Based on the above considerations, the main objectives of this research were to investigate (1) if prediction accuracy for GS varies according to various dosages of marker alleles [i.e., pseudo-diploid (A); additive tetrasomic polyploidy (B), additive-non-additive tetrasomic polyploidy (C), or both B and C together] considered in the genome-based prediction models, for (2) single environments and multiple environments (i.e., including G × E), and for (3) comparing two methods, the conventional linear GBLUP (GB) with the non-linear Gaussian kernel (GK) when used with the single-kernel genetic matrices of pseudo-diploid, additive tetrasomic polyploid, and additive-non-additive tetrasomic polyploidy, or when employing two-kernel genetic matrices in the model using the kernel from additive tetrasomic polyploidy markers, together with the kernel with additive-non-additive tetrasomic polyploidy for a single environment and multi-environments (i.e., including G × E).

## Materials and Methods

### Phenotypic Data

The multi-site experiments included 169 potato breeding clones and cultivars in Helgegården, and 256 breeding clones and cultivars in Mosslunda and nearby Umeå (Supplementary Table 1, see link https://hdl.handle.net/11529/10548617). The breeding clones are in at least the fourth generation (T_4_) of selection by Svensk potatisförädling of the Swedish University of Agricultural Sciences (Ortiz et al., 2020), while the cultivars are a sample of those released and grown in Europe during the last 200 years. Helgegården and Mosslunda are rural sites near the city of Kristianstad (56°01′46′′N 14°09′24′′E, Skåne, southern Sweden), while Umeå (63°49′30′′N 20°15′50′′E) is a city in the north of Sweden. The potato cropping season lasts about 3.5–4 months in Skåne (end of May–early September), while only 90 days in Umeå (early June–end of August). The average daily temperatures during the potato growing season vary between 12 and 18°C in the southern sites, and by 12.5–16°C in Umeå, while the average monthly precipitation amounts to 42 to 64 mm in sites near Kristianstad, and 48 to 75 mm in Umeå. The daylengths range between 11.5 h (toward harvest) and 17.5 h (about mid-growing season) in Skåne, and between 14.5 (around harvest) and ca. 21 h (at the beginning of the cropping season) in Umeå.

An incomplete block design, with two replications of 10 plants each, was the field layout for the field trials in Helgegården (13 × 13 simple lattice), Mosslunda (16 × 16 simple lattice), and Umeå (16 × 16 simple lattice). Fungicides were only used in Helgegården to avoid pests such as late blight (caused by the oomycete *Phytophthora infestans*) throughout the growing season, thus, allowing to estimate tuber yield potential at this site. Crop husbandry was used for potato farming at each site.

Total tuber yield per plot (kg), tuber weight by size (<40 mm, 40–50 mm, 50–60 mm, >60 mm; kg), and tuber flesh starch (measured as percentage based on specific gravity after harvest) were evaluated across all sites. Host plant resistance to late blight was evaluated using the area under the disease progress curve (AUDPC, Fry, 1978) in Mosslunda, while reducing sugars in the tuber flesh after harvest was determined using potato glucose strip tests (Mann et al., 1991) in Umeå.

### Genotypic Data

Leaf samples --using 4 punches for each of the 256 breeding clones and cultivars included in the experiments-- were sent to Diversity Array Technology Pty Ltd (ACT, Australia) through AgriTech---Intertek ScanBi Diagnostics (Alnarp, Sweden) for further targeted genotyping following the genotype-by-sequencing approach.^1^ The 2,000 single nucleotide polymorphisms (SNPs) used for genotyping were mostly derived from SolCAP SNPs based on chromosome positions and MAF >1 in germplasm from the Centro Internacional de la Papa (CIP, Lima, Perú) and the United States. According to Selga et al. (2021a), such a number of SNP already suffices for GEBVs without losing information. Although there were very few missing genotyping data (0.1%), one breeding clone (97) and two cultivars (‘Leyla’ and ‘Red Lady’) were not included further in the analysis because they were lacking enough SNP data.

### Computing the Genomic Relationship Matrix

We first briefly described the three different cases for codifying the molecular **X** matrix proposed by Slater et al. (2016) to be used in the genomic-enabled prediction models. Then, we defined the Bayesian linear single environment model and the multi-environment model, including the G × E using the GB and GK kernel methods.

Based on Slater et al. (2016), there are three cases for codifying the **X** matrix and thus, the type of genomic relationship matrices (Table 1). According to the authors, “there are at least two possible assumptions regarding the effect of marker allele dosage on phenotype for genomic selection. One assumption would be a pseudodiploid model, where all heterozygous genotypes have an equal effect on the genotype, and that the effects of the heterozygotes is at the midpoint of the two homozygotes.”

**TABLE 1:** Coding of the design matrix for bi-allelic single nucleotide polymorphisms (A or B alleles) in a polysomic tetraploid potato considering pseudo-diploid (A), additive tetrasomic polyploid genotypes (B), and full tetraploids including non-additive effects (after Slater et al., 2016).

Genotype	Pseudo-diploid (A)	Additive tetrasomic polyploid (B)	Full tetraploid including non-additive effects (C)				
Marker effects #	1	1	1	2	3	4	5
AAAA	0	0	1	0	0	0	0
AAAB	1	1	0	1	0	0	0
AABB	1	2	0	0	1	0	0
ABBB	1	3	0	0	0	1	0
BBBB	2	4	0	0	0	0	1

### Pseudo-Diploid (A)

In this case, the marker matrix ***X*** is constructed as indicated in Table 1 (Slater et al., 2016) for the pseudo-diploid model with a column for each of the *M* markers with 0 for AAAA, 2 for BBBB, and 1 for any other form. Hence, the linear relationship between lines *j*and*k* for the GBLUP method (GB), can be constructed as:


$$G\,B_{j\,k}=\frac{\frac{1}{M}\,\sum_{i=1}^{M}\left(x_{j\,i}-2\,p_{i}\right)\,\left(x_{k\,i}-2\,p_{i}\right)}{2\,p_{i}\,\left(1-p_{i}\right)}$$

The diagonal of matrix ***GB*** can be constructed as in Slater et al. (2016):


$$G\,B_{j\,j}=1+\frac{1}{M}\,\frac{\sum_{i=1}^{M}\left(x_{j\,i}^{2}-\left(1+2\,p_{i}\right)\,x_{j\,i}+2\,p_{i}^{2}\right)}{2\,p_{i}\,\left(1-p_{i}\right)}$$

where M is the number of markers and *p_i_* for the *i^th^* marker is computed as:


$$\frac{4\,n_{b\,b\,b\,b}+3\,n_{a\,b\,b\,b}+2\,n_{a\,a\,b\,b}+n_{a\,a\,a\,b}}{4\,N}$$

where *N* is the total number of individuals and *n*_*bbbb*_,*n*_*abbb*_,*n*_*aabb*_,*n*_*aaab*_ is the number of individuals of genotypes BBBB, ABBB, AABB, and AAAB, respectively.

To model a more complex relationship between the lines, the Gaussian kernel, defined as $\text{GK}=\exp\left(-h\,d_{i\,i^{\prime }}^{2}/q\right),$ could be used where *h* is the bandwidth parameter that controls the rate of decay of the covariance between genotypes, and *q* is the median of the square of the Euclidean distance *d*_*ii*′_=∑_*k*_(*x*_*ik*_−*x*_*i*′*k*_)^2^, which is a measure of the genetic distance between individuals based on molecular markers. The bandwidth parameter *h* was estimated based on the empirical Bayes proposed by Pérez-Elizalde et al. (2015).

### Additive Tetrasomic (B)

Following Slater et al. (2016), Additive tetrasomic is adapted for estimating the additive marker effect by accounting for the tetraploid allele dosage. In this case, ***X*** has the dimensions *N* × *M*, but the new coding is now 0, 1, 2, 3, and 4, for AAAA, AAAB, AABB, ABBB, and BBBB, respectively.

In this study, matrix ***X*** is standardized by column (mean equals to zero and variance equals to 1) as such and according to Lopez-Cruz et al. (2015):


$$\text{GB}={\text{XX}}^{{}^{\prime }}/M$$

As in the previous case, the Gaussian kernel can be constructed as $\text{GK}=e\,x\,p\,\left(-h\,d_{i\,i^{\prime }}^{2}/q\right)$.

### Full Tetrasomic Including Non-additive Effects (C)

An alternative option for coding matrix **X** according to Slater et al. (2016) is considering additive and non-additive effects in a full tetrasomic, assuming each genotype has its own effect. In this case, there are five possible effects per SNP marker (Table 1). Then the genomic relationship between individuals *j*,*k* is computed as:


$$G\,B_{j\,k}=\frac{\frac{1}{M}\,\sum_{i=1}^{M}\left(x_{j\,i}-p_{i}\right)\,\left(x_{k\,i}-p_{i}\right)}{p_{i}\,\left(1-p_{i}\right)}$$

where M is the number of markers × 5. To compute the diagonal of this matrix, we can use:


$$G\,B_{j\,j}=1+\frac{1}{M}\,\frac{\sum_{i=1}^{M}\left(x_{j\,i}^{2}-2\,p_{i}\,x_{j\,i}+p_{i}^{2}\right)}{p_{i}\,\left(1-p_{i}\right)}$$

where *p_i_* is the frequency of each genotype, i.e., the frequency in each column. The Gaussian kernel can be calculated as in the previous cases.

### Genome-Based Bayesian Regression Models

Here we consider the single-environment and multi-environment models, each combined with two methods, linear kernel GBLUP (GB), and non-linear Gaussian (GK). In addition, each of these combinations of model/method were tested with the three single-kernel methods derived from the ***X*** with marker dosage A, B, and C and the two-kernel methods combining ***X*** with marker dosage B and C.

The two-kernel method attempts to exploit the additive effects of the genomic matrix (B) and the non-additive case (C), as explained below for a single environment and multi-environments under the two kernel methods (GB and G). Thus, each of the single-environment and multiple-environment models under GB and GK had three different single-kernel methods (for A, B, and C), and one two-kernel methods (B and C).

#### Single-Environment Single-Kernel Model (Model 1)

The basic single environment model is:


(1) $$\text{y}=\mu \,1+{\text{Z}}_{g}\,\text{g}+\varepsilon$$

where ***y*** is the vector of response variables phenotypic trait, μ is an interceptor general mean, **1** is a vector of ones, the matrix ***Z_g_*** maps the phenotypic observations of the clones to the random genetic effects ***g*** with a normal distribution with mean zero and a variance-covariance structure $\sigma_{g}^{2}\text{K},N(0,\sigma_{g}^{2}\text{K}$), where $\sigma_{g}^{2}$ is the variances, and ***K*** is a relationship matrix between lines based on the marker matrix ***X***. This matrix ***K*** can be constructed with the GBLUP (**GB**) methods or with the Gaussian kernel (**GK**), considering the 3 cases for codifying as previously described (A, B, C). The random vectors of errors ε has a normal distribution with mean zero and variance $\sigma_{\varepsilon }^{2}$, $N\,\left(0,\sigma_{\varepsilon }^{2}\,\text{I}\right)$, where ***I*** is the identity matrix.

#### Single-Environment Two-Kernel Model (Model 2)

This model is similar to model 1, except that it adds the effect of B plus the effect of C


(2) $$\text{y}=\mu \,1+{\text{Z}}_{g}\,{\text{g}}_{1}+{\text{Z}}_{g}\,{\text{g}}_{2}+\varepsilon$$

where ***g*_1_** and ***g*_2_** follow a normal distribution with mean zero and variance-covariance matrices $\sigma_{g_{1}}^{2}\,{\text{K}}_{1}$, $\sigma_{g_{2}}^{2}\,{\text{K}}_{2}$, respectively, where ***K*_1_** is constructed by coding the ***X*** matrix as in B (additive tetrasomic), and ***K*_2_** is made by coding matrix ***X*** as in case C (full tetrasomic including non-additive effects).

#### Multi-Environment Single-Kernel Model Including G × E (Model 3)

The environments (***e***) could be considered as fixed effects as in Jarquín et al. (2014), Lopez-Cruz et al. (2015), and Cuevas et al. (2016) or as random effects as in Martini et al. (2020). In this study, the environmental effects are taken as random effects such that the model is denoted as


(3) $$\text{y}=\mu \,1+{\text{Z}}_{1}\,\text{e}+{\text{Z}}_{2}\,\text{g}+\text{ge}+\varepsilon$$

where vector ***y***=[***y***_1_,,***y***_*s*_]′ is the observations in each of the *s* locations or environments of size *n*, μ is a fixed effect that represents the intercept or a general mean, **1** is the vector of ones of size *n*, ***Z***_1_ is a matrix that relates the observations with the environments (or sites), and **e** is the vector of random environments of size *s* that follows a normal distribution $N(0,\sigma_{e}^{2}\text{E}$) with zero mean, variance $\sigma_{e}^{2},$ and variance-covariance matrix ***E***. Note that matrix ***E*** could be an identity, such that vector ***e*** represents the intercepts or means of each environment as is the case in this study. However, the model could be improved by means of developing a matrix based on environmental similarities (environmental covariables) and is used similar to ***E*** (Jarquín et al., 2014; Perez-Rodriguez et al., 2015). Matrix ***Z***_2_ maps the phenotypic observations of the clones or genotypes and ***g*** represents the random genetic effects assumed to have a normal distribution with mean zero and a variance-covariance structure $\sigma_{g}^{2}\text{K},N(0,\sigma_{g}^{2}\text{K}$), with $\sigma_{g}^{2}$ being the genetic variance scaled factor.

The G × E interaction random component ***ge*** is assumed to have a normal distribution with a mean vector of zero, and a structured variance-covariance $\sigma_{g\,e}^{2}\text{GE},N(0,\sigma_{g\,e}^{2}\text{GE}$), $\sigma_{g\,e}^{2}$ is the scaled G × E variance, and the ***GE*** could be estimated using the Kronecker product of their covariance ***GE***=***K***⊗***E*** (Martini et al., 2020); this implies that the data were balanced between environments, which would also be an alternative to consider the interaction between the components ***Z***_1_***e***, and ***Z*_2_*g*** by means of the Haddamar #, $\text{GE}={\text{Z}}_{1}\,{\text{EZ}}_{1}^{{}^{\prime }}\,\#\,{\text{Z}}_{2}\,{\text{KZ}}_{2}^{{}^{\prime }}$ (Jarquín et al., 2014; Martini et al., 2020). The vector of random errors **ε** is assumed to have a normal distribution with mean zero homogeneous and identical variance $\sigma_{\varepsilon }^{2}$, $N\,\left(0,\sigma_{\varepsilon }^{2}\,\text{I}\right)$, where ***I*** is the identity matrix.

As previously mentioned, matrix ***K*** can be constructed using the GBLUP (**GB**) or the Gaussian kernel (**GK**), considering each of the codification cases of matrix ***X***, as previously explained.

#### Multi-Environment Two-Kernel Model Including G × E (Model 4)

This model adds two kernels to model 3, in order to include marker dosages B and C


(4) $$\text{y}=\mu \,1+{\text{Z}}_{1}\,\text{e}+{\text{Z}}_{2}\,{\text{g}}_{1}+{\text{g}}_{1}\,\text{e}+{\text{Z}}_{2}\,{\text{g}}_{2}+{\text{g}}_{2}\,\text{e}+\varepsilon$$

where random genetic effects ***g*_1_** and ***g*_2_** follow a multivariate normal distribution, with vector of means zero and variance-covariance $\sigma_{g_{1}}^{2}\,{\text{K}}_{1}$, $\sigma_{g_{2}}^{2}\,{\text{K}}_{2}$, respectively, where ***K*_1_** is constructed using the marker dosage codes for case B (additive tetrasomic), and ***K*_2_** is constructed using the marker dosage code for case C (full tetrasomic including non-additive); the interaction terms ***g***_1_***e*** and ***g*_2_*e*** are modeled with a distribution with mean equal to zero and variance covariance matrices $\sigma_{g\,e_{1}}^{2}\,{\text{Z}}_{1}\,{\text{EZ}}_{1}^{{}^{\prime }}\,\#\,{\text{Z}}_{2}\,{\text{K}}_{1}\,{\text{Z}}_{2}^{{}^{\prime }}$ and $\sigma_{g\,e_{2}}^{2}\,{\text{Z}}_{1}\,{\text{EZ}}_{1}^{{}^{\prime }}\,\#\,{\text{Z}}_{2}\,{\text{K}}_{2}\,{\text{Z}}_{2}^{{}^{\prime }}$.

### Assessment of Genome-Based Prediction Accuracy

For the single site (models 1 and 2, Eqs. 1 and 2), we extracted 30 random samples to form groups; 70% for the training set (TRN) and 30% to be predicted (testing, TST set). For the multi-environment (models 3 and 4, Eqs. 3 and 4), we extracted four random folds each with 10 samples using a random cross-validation called CV2 that consists of predicting one line in one site, knowing the value of that line in at least one of the rest of the sites. We used Monte Carlo Markov Chain (MCMC), using the BGGE software (Granato et al., 2018), to fit the four models and to predict the individuals in the TST sets. For each of the samples, we computed the genome-based predictions and correlated them with the observed values. We reported the mean of the correlation between the predicted and the observed values and its standard deviations.

Markov Chain Monte Carlo (MCMC) diagnostics are tools that are used to investigate whether the quality of a sample generated with an MCMC algorithm is sufficient for providing an accurate approximation of the target distribution. The MCMC has diagnostic tools for testing (1) whether a large portion of the MCMC sample has been drawn from distributions that are significantly different from the target distribution or for observing (2) whether the size of the generated sample is too small. In this study in order to minimize the random error from the MCMC, we performed 30,000 iterations, with a burn-in of the first 5,000 and a thinning of 2. Supplementary Figure 1 displayed the MCMC results from the model 1 analyses of one trait; the genetic variance components are correctly mixed, achieving a correct convergence and thus, generating the posterior probability distribution.

### Data Availability

The marker data as well the phenotype data for each of the three environments are stored at the link https://hdl.handle.net/11529/10548617.

## Results

### Single-Environment Single Kernel and Two-Kernel Analyses (Models 1 and 2 With GB and GS)

**FIGURE 1:**
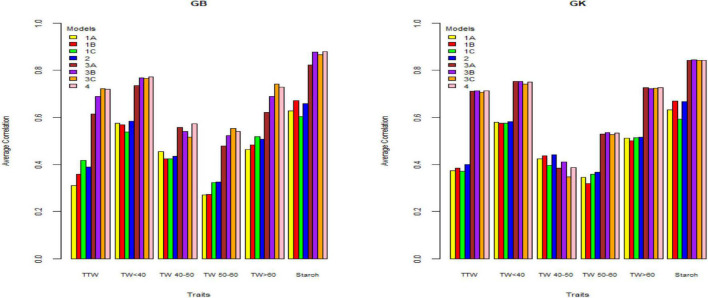
Genome-based predictions (average correlation between observed and predicted values) of potato breeding clones and cultivars in Helgegärden site for total tuber weight (TTW), tuber weight with size below 40 mm (TW < 40), tuber weight with 40–50 mm size (TW 40–50), tuber weight with 50–60 mm size (TW 50–60), tuber weight above 60 mm size (TW > 60), and tuber starch percentage (Starch) considering single environment pseudo-diploid (A) (model 1) (1A), additive tetrasomic polyploid (B) (model 1) (1B), full tetraploid (C) (model 1) (1C), and B-C (model 2) (2) and multi-environment pseudo-diploid (A) (model 3) (3A), additive tetrasomic polyploid (B) (model 3) (3B), full tetraploid (C) (model 3) (3C), and B-C (model 4). These models (1–4) combined marker matrices A, B, and C (1A, 1B, 1C, 2, 3A, 3B, 3C, and 4) were combined with linear kernel GB (GBLUP) and non-linear kernel GK (Gaussian kernel).

**FIGURE 2:**
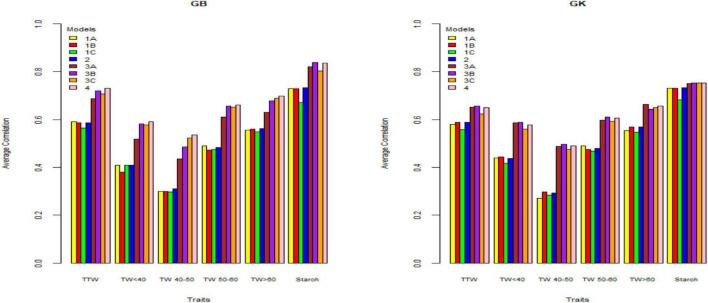
Genome-based predictions (average correlation between observed and predicted values) of potato cultivars in Mosslunda site for total tuber weight (TTW), tuber weight with size below 40 mm (TW < 40), tuber weight with 40–50 mm (TW 40–50), tuber weight with 50–60 mm size (TW 50–60), tuber weight above 60 mm size (TW > 60), and tuber starch percentage (Starch) considering single environment pseudo-diploid (A) (model 1) (1A), additive tetrasomic polyploid (B) (model 1) (1B), full tetraploid (C) (model 1) (1C), and B-C (model 2) (2) and multi-environment pseudo-diploid (A) (model 3) (3A), additive tetrasomic polyploid (B) (model 3) (3B), full tetraploid (C) (model 3) (3C), and B-C (model 4). These models (1–4) with marker matrices A, B, and C (1A, 1B, 1C, 2, 3A, 3B, 3C, and 4) were combined with linear kernel GB (GBLUP) and non-linear kernel GK (Gaussian kernel).

**FIGURE 3:**
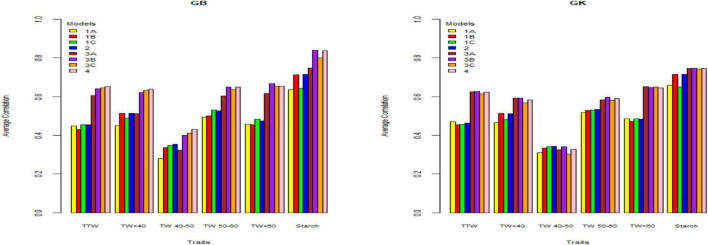
Genome-based predictions (average correlation between observed and predicted values) of potato cultivars in Umeå site for total tuber weight (TTW), tuber weight with size below 40 mm (TW < 40), tuber weight with 40–50 mm size (TW 40–50), tuber weight with 50–60 mm size (TW 50–60), tuber weight above 60 mm size (TW > 60), and tuber starch percentage (Starch) considering single environment pseudo-diploid (A) (model 1) (1A), additive tetrasomic polyploid (B) (model 1) (1B), full tetraploid (C) (model 1) (1C), and B-C (model 2) (2) and multi-environment pseudo-diploid (A) (model 3) (3A), additive tetrasomic polyploid (B) (model 3) (3B), full tetraploid (C) (model 3) (3C), and B-C (model 4). These models (1–4) with marker matrices A, B, and C (1A, 1B, 1C, 2, 3A, 3B, 3C, and 4) were combined with linear kernel GB (GBLUP) and non-linear kernel GK (Gaussian kernel).

Tables 2–4 and Figures 1–3 give the 2020 results of Helgegården, Mosslunda, and Umeå, respectively, for several traits, of the average correlation (between observed and predictive values) and their standard deviation for the single trial analysis for each of methods GBLUP (GB) and GK, considering (model 1) constructing matrix **X** as pseudo-diploid (A), additive tetrasomic polyploid (B), full tetraploid (C), and the combination of B-C (model 2).

**TABLE 2:** Single-environment genomic best linear unbiased predictor (GBLUP, GB) and Gaussian kernel (GK) prediction accuracy (±standard deviation) for potato tuber characteristics considering pseudo-diploid (A) (model 1), additive tetrasomic polyploid (B) (model 1), full tetraploid (C) (model 1), and B-C (model 2) with 30 random partitions (70% training and 30% testing) in Helgegården 2020 (*N* = 169).

Characteristic	A Pseudo-diploid (Model 1)	B Additive tetrasomic polyploidy (Model 1)	C Full tetraploid (Model 1)	B-C (Model 2)
**GB**				
Total tuber weight	0.310 ± 0.127	0.359 ± 0.131	0.418 ± 0.110	0.389 ± 0.136
Tuber weight < 40	0.576 ± 0.094	0.568 ± 0.110	0.539 ± 0.131	0.584 ± 0.113
Tuber weight 40–50	0.455 ± 0.084	0.424 ± 0.091	0.424 ± 0.094	0.434 ± 0.086
Tuber weight 50–60	0.270 ± 0.126	0.273 ± 0.129	0.324 ± 0.099	0.326 ± 0.122
Tuber weight > 60	0.464 ± 0.103	0.483 ± 0.109	0.518 ± 0.096	0.508 ± 0.107
Starch (%)	0.629 ± 0.077	0.671 ± 0.075	0.604 ± 0.094	0.658 ± 0.075
**GK**				
Total tuber weight	0.374 ± 0.121	0.389 ± 0.135	0.372 ± 0.142	0.399 ± 0.132
Tuber weight < 40	0.580 ± 0.111	0.576 ± 0.108	0.574 ± 0.119	0.582 ± 0.110
Tuber weight 40–50	0.424 ± 0.066	0.437 ± 0.082	0.395 ± 0.093	0.442 ± 0.081
Tuber weight 50–60	0.346 ± 0.111	0.318 ± 0.100	0.358 ± 0.108	0.367 ± 0.110
Tuber weight > 60	0.511 ± 0.107	0.502 ± 0.113	0.514 ± 0.111	0.516 ± 0.112
Starch (%)	0.633 ± 0.074	0.669 ± 0.076	0.592 ± 0.076	0.667 ± 0.074

**TABLE 3:** Single-environment genomic best linear unbiased predictor (GBLUP) (GB) and Gaussian kernel (GK) prediction accuracy (±standard deviation) for potato tuber characteristics and host plant resistance to late blight (measured by area under disease progress curve or AUDPC) considering pseudo-diploid (A) (model 1), additive tetrasomic polyploid (B) (model 1), and full tetraploid (C) (model 1) and B-C (model 2) with 30 random partitions (70% training and 30% testing) in Mosslund**a** 2020 (*N* = 253).

Characteristic	A Pseudo-diploid (Model 1)	B Additive tetrasomic polyploidy (Model 1)	C Full tetraploid (Model 1)	B-C (Model 2)
**GB**				
AUDPC	0.636 ± 0.065	0.613 ± 0.062	0.624 ± 0.067	0.630 ± 0.063
Total tuber weight	0.590 ± 0.059	0.587 ± 0.058	0.564 ± 0.057	0.587 ± 0.059
Tuber weight < 40	0.409 ± 0.088	0.380 ± 0.088	0.409 ± 0.076	0.409 ± 0.086
Tuber weight 40–50	0.300 ± 0.085	0.300 ± 0.086	0.298 ± 0.100	0.311 ± 0.087
Tuber weight 50–60	0.490 ± 0.079	0.472 ± 0.066	0.474 ± 0.065	0.483 ± 0.066
Tuber weight > 60	0.555 ± 0.066	0.559 ± 0.071	0.549 ± 0.063	0.562 ± 0.069
Starch (%)	0.729 ± 0.045	0.729 ± 0.049	0.672 ± 0.059	0.734 ± 0.050
**GK**				
AUDPC	0.621 ± 0.065	0.622 ± 0.062	0.624 ± 0.064	0.629 ± 0.062
Total tuber weight	0.580 ± 0.062	0.589 ± 0.057	0.557 ± 0.060	0.589 ± 0.058
Tuber weight < 40	0.440 ± 0.083	0.444 ± 0.085	0.417 ± 0.078	0.434 ± 0.080
Tuber weight 40–50	0.270 ± 0.092	0.297 ± 0.089	0.284 ± 0.083	0.292 ± 0.092
Tuber weight 50–60	0.490 ± 0.074	0.476 ± 0.066	0.468 ± 0.063	0.479 ± 0.064
Tuber weight > 60	0.553 ± 0.066	0.569 ± 0.071	0.547 ± 0.067	0.568 ± 0.071
Starch (%)	0.731 ± 0.042	0.730 ± 0.049	0.683 ± 0.052	0.734 ± 0.050

**TABLE 4:** Single-environment genomic best linear unbiased predictor (GBLUP) (GB) and Gaussian kernel (GK) prediction accuracy (±standard deviation) for potato tuber characteristics considering pseudo-diploid (A) (model 1), additive tetrasomic polyploid (B) (model 1), full tetraploid (C) (model 1), and B-C (model 2) with 30 random partitions (70% training and 30% testing) in Umeå 2020 (*N* = 252).

Characteristic	A Pseudo-diploid (model 1)	B Additive tetrasomic polyploidy (model 1)	C Full tetraploid (model 1)	B-C (model 2)
**GB**				
Total tuber weight	0.448 ± 0.078	0.431 ± 0.092	0.455 ± 0.077	0.455 ± 0.084
Tuber weight < 40	0.450 ± 0.093	0.515 ± 0.075	0.490 ± 0.075	0.514 ± 0.075
Tuber weight 40–50	0.280 ± 0.091	0.336 ± 0.097	0.348 ± 0.083	0.354 ± 0.091
Tuber weight 50–60	0.495 ± 0.085	0.500 ± 0.083	0.531 ± 0.061	0.528 ± 0.076
Tuber weight > 60	0.458 ± 0.074	0.456 ± 0.080	0.482 ± 0.058	0.474 ± 0.073
Starch (%)	0.636 ± 0.058	0.714 ± 0.038	0.642 ± 0.061	0.716 ± 0.041
Reducing sugars	0.351 ± 0.136	0.390 ± 0.133	0.351 ± 0.153	0.375 ± 0.138
**GK**				
Total tuber weight	0.471 ± 0.074	0.454 ± 0.086	0.456 ± 0.077	0.464 ± 0.086
Tuber weight < 40	0.465 ± 0.085	0.513 ± 0.075	0.480 ± 0.076	0.511 ± 0.077
Tuber weight 40–50	0.310 ± 0.080	0.335 ± 0.083	0.342 ± 0.082	0.342 ± 0.086
Tuber weight 50–60	0.519 ± 0.084	0.529 ± 0.076	0.531 ± 0.065	0.534 ± 0.074
Tuber weight > 60	0.486 ± 0.068	0.473 ± 0.079	0.485 ± 0.064	0.483 ± 0.076
Starch (%)	0.660 ± 0.048	0.716 ± 0.038	0.651 ± 0.057	0.715 ± 0.039
Reducing sugars	0.346 ± 0.131	0.387 ± 0.133	0.317 ± 0.138	0.367 ± 0.127

#### Single-Environment Analyses (Helgegården) (Models 1 and 2 With GB and GK)

Results from Helgegården (Table 2) for single-kernel model 1 for the six traits show that the pseudo-diploid structure (A), the additive polyploid structure (B), the full tetraploid including non-additive effects (C), and the two-kernels B-C model 2, gave the best prediction accuracy to starch (%) and tuber weight below 40 mm in size for both GB and GK methods (Figure 1). Starch (%) values ranged from 0.604 to 0.671 for the GB method and from 0.592 to 0.669 for the GK method, whereas the tuber weight below 40 mm in size ranged from 0.539 to 0.584 for the GB method, and from 0.574 to 0.582 for the GK method.

The additive tetrasomic additive polyploid structure (B) gave a relatively high prediction accuracy for tuber starch percentage under both methods: GB (0.671) and GK (0.669). The full tetraploid including non-additive effects (C) gave two traits the highest genome-based prediction accuracy under the GB method, i.e., total tuber weight (0.418) and tuber weight with size above 60 mm (0.518) (Table 2 and Figure 1).

The two-kernel combination of the additive and non-additive tetrasomic B and C (model 2) gave the best prediction accuracy for tuber weight with size below 40 mm (0.584) and tuber weights with 50–60 mm size (0.326). Interestingly, the combination of B and C two-kernel structure (model 2) under the Gaussian kernel (GK) gave a better prediction accuracy than the GBLUP (GS) for 4 traits, except for total tuber weight and tuber weight below 40 mm size (Table 2 and Figure 1).

#### Single-Environment Analyses (Mosslunda) (Models 1 and 2 With GB and GK)

Results from Mosslunda (Table 3) showed the traits AUDPC (which measures host plant resistance to late blight) and starch (%) as the best genome-based predicted traits for both single kernel (model 1) and two-kernel (model 2) and marker structure (A, B, C) combinations with relatively high accuracy (ranging from 0.613 to 0.734). For these two traits, methods GS and GK gave very similar prediction accuracy.

Results from Mosslunda further shows that the pseudo-diploid structure (A) gave the best prediction accuracy for the four traits under GB, AUDPC (0.636), total tuber weight (0.59), tuber weight with below 40 mm size (0.409), and tuber weight with 50–60 mm size (0.490) (also high under GK: 0.490). The additive tetrasomic additive polyploid structure (B) gave the best predictions under the GK for tuber weight traits, i.e., total tuber weight (0.589), tuber weight with size below 40 mm (0.444), tuber weight with 40–50 mm size (0.297), and tuber weight with size above 60 mm (0.569) (Figure 2).

The combination of the additive and non-additive tetrasomic B and C (model 2) gave the best prediction accuracy for the four traits under the GB method, and for the three traits under the GK method (Table 3). The best traits for the GB method were tuber weight below 40 mm size (0.409), tuber weight with 40–50 mm size (0.31), tuber weight above 60 mm size (0.562), and starch % (0.734), whereas the best traits under the GK method showed a relatively low improvement for genome-based prediction accuracy (AUDPC, 0.629; total tuber weight, 0.589; and tuber starch percentage, 0.734) (Table 3 and Figure 2).

#### Single-Environment Analyses (Umeå) (Models 1 and 2 With GB and GK)

Results from Umeå (Table 4) (that include the reducing sugar trait) showed that tuber starch (%) was the best genome-based predicted trait for both single kernel (model 1) and two-kernel (model 2) and marker structure (A, B, C) combinations with relative high accuracy (ranging from 0.636 to 0.716). For these two traits, GK methods gave slightly higher prediction accuracy than the GS method.

Results also showed that the pseudo-diploid structure (A) gave the best prediction accuracy for only the two traits under the GK methods: total tuber weight (0.471) and tuber weight above 60 mm size (0.486). The additive tetrasomic structure (B) gave the best predictions under GB for only the two traits: tuber weight below 40 mm size (0.515) and reducing sugars (0.39), whereas the GK had three traits with the highest prediction accuracy: i.e., tuber weight below 40 mm size (0.513), tuber starch percentage (0.716), and reducing sugars (0.387) (Table 4). The full tetraploid model including non-additive effects (C) did find two traits under the GB that were the best predictive traits; i.e., tuber weight 50–60 mm size (0.531) and tuber weight above 60 mm size (0.482), but only tuber trait with 40–50 mm size was predicted under the GK method (0.342) (Figure 3).

The combination of the two-kernel additive and non-additive tetrasomic B and C (model 2) gave the best prediction accuracy for the three traits under the GB method and for the two traits under the GK method (Table 4). The best traits for the GB method were total tuber weight (0.455), tuber weight with 40–50 mm size (0.354), and tuber starch percentage (0.716); whereas the best predicted traits under the GK method were tuber weight with 40–50 mm size and tuber weight with 50–60 mm size, whose prediction accuracy estimates were 0.342 and 0.534, respectively (Table 4).

### Summary of Single-Site Analyses

The results (Tables 2–4) show unclear trends in the genome-based prediction accuracy comparing the different structures of the marker matrices and the methods (GBLUP vs. Gaussian kernel) for the different traits. At Helgegården, the two-kernel combinations (model 2) show an increase in prediction accuracy for most of the traits under the GK method as compared with those obtained under A, B, and C marker structures (Figure 1). However, at Mosslunda, model 2 increased the prediction accuracy of traits under GB, as well as GK (Figure 2). For Umeå, an unclear trend of marker forms and methods were found (Figure 3); however, model 2 and model 1 under B are always the best for starch % under GB and GK.

The trait with the highest prediction accuracy for all sites under A, B, C (model 1), model 2, and for the GB and GK methods was the highly heritable tuber starch percentage. Another trait with relatively high prediction accuracy was the total tuber weight. Concerning the single-kernel (model 1) vs. the two-kernel method (model 2), evidences show an increase in prediction accuracy of the combination of two kernels (model 2) over model 1. Non-linear Gaussian kernel (GK) does not show any clear advantage over the linear kernel GBLUP (GB).

### Multi-Environment Single-Kernel and Two-Kernel Analyses (Models 3 and 4 With GB and GK)

Tables 5–7 and Figures 1–3 give the prediction results of Helgegården, Mosslunda, and Umeå, respectively, for several traits and their standard deviation for the multi-environment analyses for each of the methods (GB and GK), considering the single-kernel (model 3) for pseudo-diploid (A), additive tetrasomic polyploid (B), full tetraploid (C), and the two-kernel combination of B-C (model 4).

**TABLE 5:** Multi-environment, genomic best linear unbiased predictor (GBLUP) (GB) and Gaussian-kernel (GK) prediction accuracy (±standard deviation) for potato tuber characteristics considering pseudo-diploid (A) (model 3), additive tetrasomic polyploid (B) (model 3), full tetraploid (C) (model 3), and B-C (model 4) with fourfold partitions of 10 random samples each in Helgegården 2020 (*N* = 169).

Characteristic	A Pseudo-diploid model 3	B Additive tetrasomic polyploidy model 3	C Full tetraploid model 3	B-C Model 4
**GB**				
Total tuber weight	0.615 ± 0.090	0.688 ± 0.084	0.722 ± 0.081	0.720 ± 0.080
Tuber weight < 40	0.734 ± 0.064	0.768 ± 0.060	0.765 ± 0.058	0.772 ± 0.057
Tuber weight 40–50	0.559 ± 0.126	0.540 ± 0.125	0.516 ± 0.144	0.574 ± 0.124
Tuber weight 50–60	0.480 ± 0.108	0.523 ± 0.102	0.553 ± 0.104	0.540 ± 0.108
Tuber weight > 60	0.622 ± 0.088	0.690 ± 0.087	0.741 ± 0.072	0.738 ± 0.078
Starch (%)	0.824 ± 0.053	0.879 ± 0.038	0.867 ± 0.042	0.880 ± 0.036
**GK**				
Total tuber weight	0.711 ± 0.078	0.713 ± 0.079	0.707 ± 0.082	0.714 ± 0.081
Tuber weight < 40	0.752 ± 0.061	0.752 ± 0.061	0.741 ± 0.061	0.750 ± 0.060
Tuber weight 40–50	0.386 ± 0.168	0.410 ± 0.159	0.346 ± 0.155	0.387 ± 0.154
Tuber weight 50–60	0.529 ± 0.107	0.536 ± 0.105	0.526 ± 0.105	0.534 ± 0.106
Tuber weight > 60	0.726 ± 0.080	0.722 ± 0.078	0.725 ± 0.073	0.727 ± 0.074
Starch (%)	0.843 ± 0.058	0.844 ± 0.058	0.843 ± 0.058	0.844 ± 0.058

**TABLE 6:** Multi-environment, genomic best linear unbiased predictor (GBLUP), and Gaussian-kernel prediction accuracy (± standard deviation) for potato tuber characteristics considering pseudo-diploid (A) (model 3), additive tetrasomic polyploid (B) (model 3), full tetraploid (C) (model 3), and B-C (model 4) with fourfold partitions of 10 random samples each in Mosslunda 2020 (*N* = 253).

Characteristic	A Pseudo-diploid model 3	B Additive tetrasomic polyploidy model 3	C Full tetraploid model 3	B-C model 4
**GB**				
Total tuber weight	0.688 ± 0.051	0.721 ± 0.045	0.708 ± 0.056	0.730 ± 0.049
Tuber weight < 40	0.518 ± 0.104	0.581 ± 0.094	0.577 ± 0.092	0.590 ± 0.091
Tuber weight 40–50	0.435 ± 0.105	0.485 ± 0.097	0.523 ± 0.085	0.534 ± 0.086
Tuber weight 50–60	0.609 ± 0.055	0.656 ± 0.054	0.651 ± 0.058	0.662 ± 0.056
Tuber weight > 60	0.631 ± 0.062	0.679 ± 0.050	0.689 ± 0.054	0.697 ± 0.051
Starch (%)	0.820 ± 0.048	0.838 ± 0.044	0.804 ± 0.056	0.835 ± 0.047
**GK**				
Total tuber weight	0.652 ± 0.084	0.656 ± 0.082	0.623 ± 0.079	0.650 ± 0.081
Tuber weight < 40	0.587 ± 0.084	0.588 ± 0.083	0.560 ± 0.090	0.577 ± 0.085
Tuber weight 40–50	0.489 ± 0.092	0.497 ± 0.090	0.474 ± 0.086	0.489 ± 0.087
Tuber weight 50–60	0.597 ± 0.072	0.609 ± 0.069	0.592 ± 0.072	0.605 ± 0.071
Tuber weight > 60	0.664 ± 0.072	0.644 ± 0.072	0.650 ± 0.074	0.655 ± 0.070
Starch (%)	0.750 ± 0.074	0.754 ± 0.074	0.752 ± 0.076	0.752 ± 0.074

**TABLE 7:** Multi-environment, genomic best linear unbiased predictor BLUP (GBLUP), and Gaussian-kernel prediction accuracy (±standard deviation) for potato tuber characteristics considering pseudo-diploid (A) (model 3), additive tetrasomic polyploid (B) (model 3), full tetraploid (C) (model 3), and B-C (model 4) with fourfold partitions of 10 random samples each in Umeå 2020 (*N* = 252).

Characteristic	A Pseudo-diploid model 3	B Additive tetrasomic polyploidy model 3	C Full tetraploid model 3	B-C model 4
**GB**				
Total tuber weight	0.605 ± 0.074	0.642 ± 0.062	0.645 ± 0.060	0.652 ± 0.060
Tuber weight < 40	0.512 ± 0.092	0.622 ± 0.077	0.633 ± 0.071	0.639 ± 0.075
Tuber weight 40–50	0.322 ± 0.088	0.399 ± 0.098	0.411 ± 0.099	0.430 ± 0.097
Tuber weight 50–60	0.604 ± 0.074	0.650 ± 0.056	0.637 ± 0.061	0.650 ± 0.059
Tuber weight > 60	0.618 ± 0.068	0.668 ± 0.075	0.654 ± 0.085	0.654 ± 0.084
Starch (%)	0.749 ± 0.052	0.841 ± 0.035	0.802 ± 0.049	0.838 ± 0.036
**GK**				
Total tuber weight	0.625 ± 0.064	0.628 ± 0.062	0.616 ± 0.064	0.624 ± 0.063
Tuber weight < 40	0.592 ± 0.086	0.594 ± 0.088	0.569 ± 0.089	0.584 ± 0.089
Tuber weight 40–50	0.325 ± 0.108	0.340 ± 0.100	0.303 ± 0.111	0.327 ± 0.106
Tuber weight 50–60	0.585 ± 0.075	0.598 ± 0.072	0.582 ± 0.071	0.590 ± 0.071
Tuber weight > 60	0.651 ± 0.088	0.648 ± 0.088	0.649 ± 0.091	0.646 ± 0.090
Starch (%)	0.745 ± 0.067	0.746 ± 0.070	0.745 ± 0.070	0.746 ± 0.070

#### Multiple-Environment Analyses (Helgegården) (Models 3 and 4 With GB and GK)

Results from multi-environments for the prediction accuracy of the potato genotypes in Helgegården (Table 5) for the six traits show, in general, an important increase in prediction accuracy for all traits for A, B, C (model 3), and model 3 as compared with the results of the single-environment analyses. The single-kernel model 1 for the six traits showed that the pseudo-diploid structure (A), the additive polyploid structure (B), the full tetraploid including non-additive effects (C), and the two-kernels B-C model 2 gave the best prediction accuracy to starch (%), and tuber weight below 40 mm size for both GB and GK methods (Figure 1). Tuber starch (%) prediction accuracy ranged between 0.820 and 0.880, whereas tuber weight below 40 mm size predictive values ranged from 0.734 to 0.772 for both GB and GK methods.

The pseudo-diploid structure (A) gave the best prediction accuracy for only one trait (tuber weight with 40–50 mm size) under GK (0.752). The additive tetrasomic additive polyploid structure (B) showed a relatively high prediction accuracy for four traits under the GK method, tuber weight below 40 mm size (0.752), tuber weight with 40–50 mm size (0.410), tuber weight with 50–60 mm size (0.536), and tuber starch percentage (0.844) (Figure 1).

Model 3 (C) gave only three traits with the highest genome-based prediction accuracy under the GB method: total tuber weight (0.722), tuber weight with 50–60 mm size (0.553) and tuber weight with above 60 mm size (0.741) (Table 5). The two-kernel (model 4) gave the best prediction accuracy for several traits under the GB and GK methods with relatively high prediction for the four traits under the GB method (tuber weight below 40 mm size, 0.772; tuber weight with 40–50 mm size, 0.574; and tuber starch percentage, 0.880), and for the three traits under the GK method (total tuber weight, 0.714; tuber weight above 60 mm size, 0.727, and tuber starch percentage, 0.844).

#### Multiple-Environment Analyses (Mosslunda) (Models 3 and 4 and GB and GK)

Results from Mosslunda (Table 6 and Figure 2) show that for single-kernel model 1 for the six traits, the pseudo-diploid structure (A), the additive polyploid structure (B), the full tetraploid including non-additive effects (C), and the two-kernels B-C model 2 gave the best prediction accuracy to starch (%) and total tuber weight for both the GB and GK methods. However, while starch (%) prediction accuracy was around 0.750 for the GK method and 0.800 for the GB method, total tuber weight predictive values ranged from 0.688 to 0.772 for GB, and from 0.623 to 0.656 for the GK method.

Other results from Mosslunda show that model 1 with pseudo-diploid structure (A) gave the best prediction accuracy for only tuber weight above 60 mm for GK (0.664). The additive tetrasomic polyploid structure (B) model 3 gave the best prediction accuracy under the GK for all the traits except tuber weight above 60 mm size with the average correlation ranging from 0.497 (for tuber weight with 40–50 mm size) to 0.754 (for tuber starch percentage). Model 4 gave the best prediction accuracy for five traits under the GB method and a relative high prediction accuracy under the GB method (Table 6): i.e., total tuber weight (0.730), and tuber weights below 40 (0.590), 40–50 (0.540), 50–60 (0.662), and above 60 (0.697) mm sizes.

#### Multi-Environment Analyses (Umeå) (Models 3 and 4)

Results from Umeå (Table 7 and Figure 3) show that the best predicted traits were starch (%) and tuber weight above 60 mm size for both GB and GK methods with, in general, higher prediction accuracy of GB over the GK method. Evidence indicates that a single-kernel model 3 with the pseudo-diploid structure (A) gave the best prediction accuracy for only tuber weight above 60 mm size when using GK (0.651). Similar to Mosslunda, the predictions from Umeå under the multi-environment single-kernel model 3 analyses show that the additive tetrasomic additive polyploid structure (B) gave the best prediction accuracy under the GB and GK methods for several traits.

For GB, the best predictive traits were tuber weight with 50–60 mm size (0.65), tuber weight above 60 mm size (0.668), and tuber starch percentage (0.841), whereas under model 3, the five traits of GK had the highest correlation with average correlations ranging from 0.34 (for tuber weight with 40–50 mm size) to 0.746 (for tuber starch percentage). Model 4 gave the best prediction accuracy for four traits under the GB method with a relatively high prediction accuracy (Table 7) for total tuber weight (0.652), and for tuber weights below 40 (0.639), 40–50 (0.430), and 50–60 (0.650) mm sizes.

### Summary of Multi-Environment Analyses

In general, results including G × E interaction in the multi-environment analyses exploit the information on the relationship between the location-year combinations and had prediction accuracy estimates higher than those obtained from the single-environment analyses (results from models 1 and 2 vs. models 3 and 4) (Figures 1–3). For the three sites, two-kernel model 4 with GB seems to be the best method in combination with the additive tetrasomic polyploidy structure B for predicting most of the tuber traits. Most of the traits gave relatively high prediction accuracy under this combination of marker structure (A, B, C, and B-C) and the combined methods of GB and GK, including the multi-environment with the G × E model.

#### Top Performing Breeding Clones and Cultivars as per Their Genomic Best Linear Unbiased Predictions

A 3% threshold (or a selection intensity *i* of 2.268; Falconer and Mackay, 1996) was used for defining the top performing potato germplasm according to their GBLUPs at each site for both total tuber weight and tuber starch percentage, as well as AUDPC for late blight in Mosslunda, and reducing sugars in Umeå. None of the breeding clones nor the cultivars were at the top for tuber weight across sites, while starch cultivars’ ‘Serum Star’ and ‘Saprodi’ were in the top 3% for tuber starch percentage across sites. Only one breeding clone for crisps (107) had a high GBLUP for tuber starch percentage under the long day length of Umeå. Another known starch cultivar (‘Nofy’) also had a high GBLUP for this trait but below the selection intensity of 2.268 in the high tuber yielding site (Helgegården) and in Umeå. This starch cultivar, which shows a host plant resistance to late blight, and seven breeding clones (1402009, 1342004, 1410005, 1402001, 1314013, 1314015, and 1419006) had the best GBLUPs for AUDPC in Mosslunda, while the only other breeding clone for crisps (121) was within the 3% of top GBLUPs for reducing sugars in Umeå. The other seven were the released cultivars and mostly from Scandinavia.

The top 3% for tuber weight in Helgegården were five breeding clones (0101011, 0003022, 1201001, 1209001, and 2-IV-4) and the cultivar ‘Kingsman.’ Four of these breeding clones (except 2-IV-4), along with two other breeding clones (1429006 and 1415001), plus two cultivars (‘Galactica’ and ‘7FOUR7’), were at the top as per their GBLUP for tuber weight in Umeå, while 2-IV-4 along with another five breeding clones (1415001, 1402009, 1429006, 1314015, and 2-IV-6), and two cultivars (‘Papageno’ and ‘Connect’) were within the 3% threshold in Mosslunda. One of these breeding clones (1402009) was also among the top GBLUPs for tuber weight in Umeå.

There was not a single breeding clone or cultivar that had the best 3% GBLUPs for all four traits, though most of the top performing were breeding clones. Such results highlight the importance of adaptability for performing under stress; they also show that breeding for the target population of environments yields more outstanding germplasm, as shown by the high number of breeding clones for both productivity (or total tuber weight) and host plant resistance to late blight (as measured by the AUDPC), despite being from a small population size (49: 41 for table and 8 for crisps), *vis-á-vis* the number of released cultivars (207) included in the trials.

The old breeding clone SW 93-1015 (showing host plant resistance to late blight) was the female parent of 0101011, 0003022, and 1209001, while 2-IV-4 and 2-IV-6 are full sibs derived from breeding populations involving wild *Solanum* species. Breeding clones 1201001, 1314015, 1415001, and 1429006 with high GBLUPs for tuber weight were significantly above the GBLUPs of their cultivar parents’ ‘Fontane,’ ‘Carolus,’ ‘Solist,’ and ‘Arielle,’ respectively. Likewise, breeding clones 1314013 and 1314015 are full sibs and both are half sibs of 1342004 and 1419006 because they share the cultivar ‘Carolus’ as a parent. All of them had a high GBLUP for host plant resistance to late blight (and above that of their cultivar parent), as well as the full sibs 1402001 and 1402009 (derived from crossing an old breeding clone with the cultivar ‘Satina’).

## Discussion

The greatest prediction accuracy was for the starch content and host plant resistance to late blight, which were the characteristics with highest broad-sense heritability in the training population (Ortiz et al., 2021). Total tuber weight and according to sizes, as well as reducing sugars in tuber flesh, had a lower prediction accuracy and a broad-sense heritability than starch content and host plant resistance to late blight. Our research confirms the preliminary results regarding a specific gravity or an increasing trait heritability in an environment that facilitates trait scoring in the field for the host plant resistance to scab caused by a few *Streptomyces* species (Ortiz et al., 2020).

The prediction of breeding values uses an hypothesis-independent approach to account for all the quantitative genetic variation (thereby “capturing” small effects of loci) and estimates marker-allelic effects in a population. For further advancing the genomic prediction in a polysomic polyploid crop such as potato, we sought answers related to how prediction accuracy may be affected by using various dosages of marker alleles, or a single and multi-environment G × E in linear (GBLUP or GB) or non-linear (GK) models, which are further described below. We are also investigating the effect of heterozygosity in genomic prediction in potato, which suffers significantly from inbreeding depression (Golmirzaie et al., 1998a,b). Genotyping and field trials are underway for comparing hybrid (S_0_) and first generation selfing (S_1_) offspring derived from crossing cultivars with different GEBV for various characteristics.

The ensuing knowledge from ours, along with other previous research on what training set to use (Selga et al., 2021b) or number of markers to include in modeling (Selga et al., 2021a), allows improving the approach for predicting breeding values for selection, thus, making accurate and cheap modern potato crossbreeding, e.g., by selecting the most promising parents for further pairing and reducing cost for field progeny testing. Genomic prediction of breeding values may also improve the accuracy of field trials and prompt the reorganization of genetic improvement programs (Desta and Ortiz, 2014). Likewise, GEBV may facilitate an early recurrent selection in potato breeding by selecting the most promising offspring for further intermating, and particularly, for characteristics that are difficult to measure.

### Single-Environment vs. Multi-Environment G × E Genome-Based Prediction Models

In general, genome-based prediction accuracy obtained in this potato study, using different marker similarity matrices accounting for additive and non-additive marker relationship under single-environment and multi-environment models, show prediction accuracy patterns similar to those found in other studies using other species with different levels of ploidy. The process of borrowing information from multi-environment trial analyses modeling G × E provides a very useful increase of genomic-enabled prediction accuracy over the evidence obtained from the single-environment analyses. This increase in prediction accuracy of G × E models has been clearly and extensively documented, among others, in Burgueño et al. (2012), Jarquín et al. (2014), Crossa et al. (2017, 2019), and Cuevas et al. (2017, 2018, 2019) where the genomic similarity between cultivars is increased when modeling the phenomenon of G × E. That is, the appropriate statistical modeling of G × E allows borrowing information from correlated environments to the predictions of unobserved phenotypes in environments. For all the agronomy traits included in this study, the important increase in prediction accuracy when genomic prediction models include G × E models is clear. Supplementary Table 2 shows the relatively high and positive phenotypic correlations between the three sites and the six traits included in this study that explain part of the increase in genomic based prediction achieved by models including G × E as compared with the single trait models.

### Differences Between Random Cross-Validation 1 and Single-Site

Two type of random cross-validation are usually employed for comparing different models and methods. Burgueño et al. (2012) and Lopez-Cruz et al. (2015) distinguished a random cross-validation 1 (CV1) when predicting lines that were never evaluated in any environment, and random cross-validation 2 (CV2) that consists of some lines tested in only some environments but not in others. Extensive results from Burgueño et al. (2012), Jarquín et al. (2014), and Lopez-Cruz et al. (2015) demonstrated that a genome-based prediction accuracy obtained from CV1 are similar to those obtained when using a single environment (site) genome-based prediction model. Lopez-Cruz et al. (2015) mentioned that “This feature of the M × E model can be exploited in prediction problems such as CV2; however, such borrowing of information within line is not possible in CV1 and, consequently, the M × E model performs similarly to the stratified analysis for prediction of performance of lines that have no phenotypic records.”

To investigate the results of CV1 in the this study we have assessed the genomic-enabled prediction accuracy of the trait tuber weight, using the full tetrapoid (C) GBLUP kernel with model 1 (single-site), and compared it with the G × E model 3 (multi-site). The results were similar for the three sites. For site Helgegarden, model 1 gave an average genomic prediction accuracy of 0.418, whereas model 3 gave an average prediction correlation of 0.433. Similar for site Mosslunda, model 1 gave an average prediction accuracy of 0.563 vs. 0.568 as mean prediction accuracy for model 3, whereas for site Umea, the mean genomic-enabled prediction for model 1 was 0.455 and for G × E model 3 model 3 was 0.450.

This is explained by the exchange of information (borrowing of information) that is achieved in the main effects component, that is, **Z_2g_**, where **g** had the effect of each line that are predicted throughout the environments. For G × E model 3 (multi-site), borrowing (exchanging) information between lines occurs only if the lines have genetic and environmental similarity. These results showing a similar genome-based prediction accuracy between model 1 and CV1 vs. G × E model 3 (multi-site) are similar (with small differences between models) to those shown by Lopez-Cruz et al. (2015; Tables 5–7).

### Kernel Methods Under Different Potato Autopolyploid Genomic Similarity Matrices With Multi-Environment G × E Models

The use of Gaussian kernels has been extensively documented in genome-based studies as a non-linear kernel that increases prediction accuracy over the linear kernel given by the linear additive GBLUP. The non-linear kernels included in multi-environment G × E models have been shown to increase the genome-based prediction accuracy by around 5–10% in several studies (Cuevas et al., 2016, 2017, 2018, 2019; Crossa et al., 2019).

As noted in our study, including the GK in the multi-environment G × E model did not overcome the genome-based prediction accuracy over the GB method. One of the reasons could be that the some of the marker structures employed, like the full tetraploid, already account for the additive and non-additive structure of the markers; thus, no extra benefit is obtained by including models that will exploit these cryptic and small epistatic inter-locus interactions between markers. Another possibility is that the GK is not able to capture the residual epistatic interactions that may exist in the tetrasomic polyploid potato even after the use of a similarity marker structure that considers the linear additive kernel and the non-linear kernel of the full tetraploid. The two kernels (B and C), with the multi-environment G × E model using the linear GBLUP (GB) kernel, seem to capture most of the potential marker epistatic interactions without the need to add the non-linear GK kernel. More research is required in this area.

### Prediction Accuracy of Other Genomic-Enabled Predictions of Potato

Table 8 provides an up-to-date summary information on all available journal articles regarding prediction accuracy estimates of GEBV for selection in potato. This table only includes traits that were evaluated in our research: i.e., tuber weight (total and by size), host plant resistance to late blight, tuber starch percentage, and crisp quality. The prediction accuracy estimates for tuber weight and tuber starch percentage are equal or above those available in the literature, or within the known ranges for both the host plant resistance to late blight (with a bias toward high correlations) and the crisp quality as measured by reducing sugars. These are encouraging results because they show that the multi-trait, multi-environment modeling of the GEBV increased the prediction accuracy estimates, which may also vary according to training population size and type, trial data quality, and method or model use.

**TABLE 8:** Prediction accuracy (ρ) ranges of breeding values for selection of host plant resistance to late blight, tuber yield, starch percentage, and crisp quality in potato using different training population sizes and varying number of testing environments.

Characteristic	Training population size (N) and testing environments	Prediction method	ρ	References
Host plant resistance to late blight	*N* = 273; early and advanced breeding clones with records from 7 years at single site using non-replicated plots or three 4-hill plots in randomized complete block design (RCBD)	Bayesian ridge regression (BRR), Bayes B	0.24–0.31	Enciso-Rodriguez et al., 2018
	*N* = 184; breeding clones and parents with data scoring over 3 years at single site	GBLUP, Bayes A, Bayes Cπ, Bayesian LASSO (BL)	0.32–0.86	Stich and Van Inghelandt, 2018
	*N* = 241–336 at two sites using RCBD	GBLUP	0.52–0.68	Gemenet et al., 2020
	*N* = 92; first generation (T_1_) plus 4 ancestors with data scoring from 1 year at single site	BRR, Bayes A, Bayes B, Bayes C, BL	0.13–0.24	Selga et al., 2021a
	*N* = 301; two first generation (T_1_) half-sib offspring (*n*_1_ = 151, *n*_2_ = 149) with data from 1 year at single site	BRR	0.16–0.31	Selga et al., 2021b
Total tuber weight	*N* = 190; EU released cultivars evaluated in single site over 2 years using RCBD with three replications	BL, RKHS, Bayes A, Bayes B, Bayes C	ca. 0.25–ca. 0.34	Habyarimana et al., 2017
	*N* = 184; breeding clones and parents with data recording over 3 years at single site	GBLUP, Bayes A, Bayes Cπ, BL	0.43–0.55	Stich and Van Inghelandt, 2018
	*N* = 571; data from breeding clones over 6 years	GBLUP	0.06–0.31	Endelman et al., 2018
	*N* = 413; T_3_ offspring in replicated trials along with standard cultivars	HBLUP (pedigree, phenotypic, and genomic information)	0.32–0.34	Sood et al., 2020
	*N* = 241–336 at two sites using augmented designs	GBLUP	0.16–0.38	Gemenet et al., 2020
	*N* = 665, non-replicated trials of T_1_ (*n*_1_ = 465 in 4-plant plots) and T_2_ (*n*_2_ = 138 in 10-plant plots) at one site plus T_3+_ (*n*_1_ = 62 in 20-plant plots) offspring in replicated trials across three sites	BRR	0.05–0.75	Selga et al., 2021b
	*N* = 147; cultivars and very advanced breeding clones with testing across three sites over 2 years	GBLUP, BL, Bayes A, Bayes Cπ	0.55–0.59	Wilson et al., 2021
Tuber starch or specific gravity	*N* = 762; hybrid offspring derived from biparental crossing of 18 plus unrelated 74 breeding clones for model validation	GBLUP, Bayes A, Bayes C	0.09–0.81	Sverrisdóttir et al., 2017
	*N* = 190; EU released cultivars evaluated in single site over 2 years using RCBD with three replications	BL, RKHS, Bayes A, Bayes B, Bayes C	ca. 0.13–ca. 0.69	Habyarimana et al., 2017
	*N* = 1,146; mapping population (*n*_1_ = 762) over 2 years at one site (non-replicated in year 1 and RCBD with two reps in year 2) plus two testing panels (*n*_2_ = 92 incl. 18 parents of mapping population; *n*_3_ = 292 breeding clones) with trial data over years	GBLUP	0.37–0.71 (across pops) 0.75–0.83 (cross validating)	Sverrisdóttir et al., 2018
	*N* = 571; data from breeding clones over 6 years	GBLUP	0.13–0.63	Endelman et al., 2018
	*N* = 184; breeding clones and parents with data recording over 3 years at single site	GBLUP, Bayes A, Bayes Cπ, Bayesian Lasso	0.51–0.83	Stich and Van Inghelandt, 2018
	*N* = 200; non-replicated T_2_ (*n*_2_ = 138 in 10-plant plots) at one site plus T_3+_ (*n*_1_ = 62 in 20-plant plots) offspring in replicated trials across three sites	BRR	0.43–0.62	Selga et al., 2021b
	*N* = 147; cultivars and very advanced breeding clones with testing across three sites over 2 years	GBLUP, BL, Bayes A, Bayes Cπ	0.72–0.76	Wilson et al., 2021
Crisp quality (reducing sugars or fry color)	*N* = 762; hybrid offspring derived from biparental crossing of 18 plus unrelated 74 breeding clones for model validation	GBLUP, Bayes A, Bayes C	0.16–0.56	Sverrisdóttir et al., 2017
	*N* = 571; data from breeding clones over 6 years	GBLUP	0.40–ca. 0.45	Endelman et al., 2018
	*N* = 555 breeding clones evaluated in 20-plant plots in a single year	rrBLUP, Bayesian A, Bayesian Lasso, Random Forest	0.11–0.77 (of the field) 0.24–0.66 (after low temperature storage)	Byrne et al., 2020
	*N* = 1,146; mapping population (*n*_1_ = 762) over two years at one site (non-replicated in year 1 and RCBD with two reps in year 2) plus two testing panels (*n*_2_ = 92 incl. 18 parents of mapping population; *n*_3_ = 292 breeding clones) with trial data over years	GBLUP	0.28–0.48 (across pops) 0.39–0.79 (cross validating)	Sverrisdóttir et al., 2018

## Conclusion

The results for single-site analyses of genome-based prediction accuracy comparing the different structures of the marker matrices and the methods (GBLUP vs. Gaussian kernel) for the different traits show that the trait with the highest prediction accuracy for one kernel on marker structures A, B, C (model 1), and for two-kernel (model 2) and for linear GB kernel and non-linear GK kernel was tuber starch percentage, followed by total tuber weight. Regarding single kernel (model 1) vs. the two-kernel method (model 2), results show an increase in prediction accuracy of the combinations of two kernels (model 2) over model 1. Furthermore, GK does not show any clear advantage over the linear kernel GB. In general, results including G × E interaction in the multi-environment analyses had prediction accuracy estimates higher than those obtained from the single-environment analyses. Two-kernel model 4 for multi-environment models with linear kernel GB is the best combination. Most of the traits gave relatively high prediction accuracy under this combination of marker structure (A, B, C, and B-C), methods GB and GK, including the multi-environment with G × E model.

## Data Availability Statement

The datasets presented in this study can be found in online repositories. The names of the repository/repositories and accession number(s) can be found below: https://hdl.handle.net/11529/10548617, Dataverse.

## Author Contributions

RO, FR, and JCr contributed to the conceptualization and field-testing layouts. FR contributed to the field data recording. JCr, JCu, and PP-R contributed to the methodology for data analysis. RO, JCr, and JCu wrote the first manuscript draft. RO, FR, JCr, JCu, and PP-R reviewed and edited the draft. RO was responsible for project grants acquisition and management. All authors have read and agreed to the final version of the manuscript.

## Conflict of Interest

The authors declare that the research was conducted in the absence of any commercial or financial relationships that could be construed as a potential conflict of interest.

## Publisher’s Note

All claims expressed in this article are solely those of the authors and do not necessarily represent those of their affiliated organizations, or those of the publisher, the editors and the reviewers. Any product that may be evaluated in this article, or claim that may be made by its manufacturer, is not guaranteed or endorsed by the publisher.

## Footnotes

1
https://www.diversityarrays.com/technology-and-resources/targeted-genotyping/
