# Supplementary material for: Genome-Based Genotype × Environment Prediction Enhances Potato (Solanum tuberosum L.) Improvement Using Pseudo-Diploid and Polysomic Tetraploid Modeling
Source: Front Plant Sci. 2022 Feb 7;13:785196. doi: 10.3389/fpls.2022.785196 (PMC8859116; doi:10.3389/fpls.2022.785196)
Supplement: Supplementary file 1 [file Data_Sheet_1.pdf]

Table S1. SLU's Svensk potatisförädling breeding clones and cultivars available for growing in Sweden used for field trials in Helgegården, Mosslunda and Umeå.

Cultivar or Breeding Clone	Country	Released or Crossing Year
Belinda	AUT	1979
Ditta	AUT	1989
Kerkauer Kipfler	AUT	N/A
Linzer Delikatess	AUT	1975
Afra	BRD	1990
Agila	BRD	2006
Agria	BRD	1985
Alexandra	BRD	2008
Allians	BRD	2003
Almonda	BRD	2011
Annalena	BRD	N/A
Antonia	BRD	2008
Baby Lou	BRD	N/A
Bellarosa	BRD	2004
Belmonda	BRD	2010
Birgit	BRD	2009
Blaue Annelise	BRD	N/A
Centifolia	BRD	1919
Cilena	BRD	1981
Concordia	BRD	2008
Corinna	BRD	N/A
Edison	BRD	N/A
Finka	BRD	2001

Gala	BRD	2002
Goldmarie	BRD	N/A
Granola	BRD	1975
Gunda	BRD	1999
Hansa	BRD	1957
Heiderot	BRD	1977
Jelly	BRD	2002
Jule	BRD	N/A
Julinka	BRD	N/A
Krone	BRD	2002
Laura	BRD	1998
Lea	BRD	N(A
Leyla	BRD	1988
Lilly	BRD	N/A
Linda	BRD	1974
Ludmilla	BRD	2008
Marena	BRD	1995
Merle	BRD	N/A
Nandina	BRD	2010
Natalia	BRD	N/A
Natascha	BRD	2006
Nicola	BRD	1973
Odenwälder Blaue	BRD	1908
Orla	BRD	2001
Otolia	BRD	N/A
Papageno	BRD	2019
Pocahontas	BRD	N/A

Princess	BRD	1998
Quarta	BRD	1979
Queen Anne	BRD	2012
Ragna	BRD	1982
Ramona	BRD	1988
Red Emmalie	BRD	N/A
Red Fantasy	BRD	2006
Red Lady	BRD	2004
Regina	BRD	2009
Rodriga	BRD	2005
Rosara	BRD	1990
Rossini	BRD	N/A
Salad Blue	BRD	N/A
Selma	BRD	1972
Sieglinde	BRD	1935
Solara	BRD	1989
Solist	BRD	1999
Sunshine	BRD	N/A
Talent	BRD	2006
Torenia	BRD	N/A
Venezia	BRD	2009
Verdi	BRD	2003
Anuschka	BRD	2004
Belana	BRD	2000
Quadriga	BRD	2005
Blaue St. Galler	CH	N/A
Madeira	CH	N/A

Adretta	DDR	1975
Karlena	DDR	1988
Aeggeblomme	DK	N/A
Folva	DK	1989
Tammiston	FIN	1930
Timo	FIN	1975
Altesse	FRA	2000
Amandine	FRA	1993
Anais	FRA	1997
Aura	FRA	1951
Belle de Fontenay	FRA	1885
Blue Belle	FRA	2008
Charlotte	FRA	1981
Cherie	FRA	1997
Cheyenne	FRA	N/A
Dalida	FRA	N/A
Gaiane	FRA	2018
Glorietta	FRA	2020
Gwenne	FRA	2011
Juliette	FRA	1997
La Ratte	FRA	1965
Maestro	FRA	2001
Marine	FRA	1994
Monique	FRA	2020
Vitelotte	FRA	1815
Yona	FRA	2008
Zoe	FRA	2018

Anya	GB	1996
Arran Pilot	GB	1930
Arran Victory	GB	1918
British Queen	GB	1984
Casa Blanca	GB	2010
Emily	GB	N/A
Golden Wonder	GB	1906
Highland Burgundy Red	GB	1930
Inca Bella	GB	2010
International Kidney	GB	1879
King Edward	GB	1902
Kingsman	GB	N/A
Maris Bard	GB	1972
Maris Peer	GB	1962
Marys Rose	GB	2001
Mayan Gold	GB	2001
Mayan Rose	GB	N/A
Pink Fir Apple	GB	1850
Red Duke of York	GB	N/A
Rocket	GB	1987
Sarpo Una	GB	2010
Shetland Black	GB	N/A
Swift	GB	1994
Valor	GB	1993
Foxton	GB	1981
Sarpo Mira	HUN	2003
Cara	IRL	1973

Colleen	IRL	1991
Galactica	IRL	2003
Rooster	IRL	1990
Setanta	IRL	2004
Raudar	IS	1800
7 FOUR 7	NDL	2014
A	NDL	2018
Allstar	NDL	2015
Alouette	NDL	2014
Annabelle	NDL	2001
Anouk	NDL	2014
Apache	NDL	1986
Arielle	NDL	1999
Arrow	NDL	2004
Arsenal	NDL	2009
Asperges	NDL	N/A
Asterix	NDL	1991
Avenue	NDL	2010
Bintje	NDL	1910
Bionica	NDL	2008
Carolus	NDL	2012
Challenger	NDL	2008
Colomba	NDL	2011
Connect	NDL	2012
Dartiest	NDL	2012
Desiree	NDL	1962
Eigenheimer	NDL	1885

Fontane	NDL	1999
Frieslander	NDL	1990
Hind	NDL	2018
Inova	NDL	1999
Jazzy	NDL	2010
Kuras	NDL	1996
Labella	NDL	2008
B	NDL	2019
C	NDL	2007
D	NDL	2010
E	NDL	2008
Lady Christl	NDL	1996
F	NDL	1996
G	NDL	1988
Laperla	NDL	2010
Marabel	NDL	1993
Marilyn	NDL	2006
Masai	NDL	2014
H	NDL	2001
Minerva	NDL	1988
Montana	NDL	1998
Monte Carlo	NDL	2009
I	NDL	2007
Nofy	NDL	2017
J	NDL	2007
Purple Rain	NDL	2019
Record	NDL	1932

Saprodi	NDL	2014
Serum Star	NDL	N/A
Sevilla	NDL	2018
K	NDL	2005
Sunita	NDL	2014
Taisiya	NDL	2011
Tarzan	NDL	1989
Theresa	NDL	1979
Triplo	NDL	2000
Twister	NDL	2017
Ultra	NDL	1999
Marius	NOR/POL	1893
0003022	SLU	2000
0101011	SLU	2001
0502047	SLU	2005
1201001	SLU	2012
1209001	SLU	2012
1211009	SLU	2012
1212001	SLU	2012
1213008	SLU	2012
1312011	SLU	2013
1314013	SLU	2013
1314015	SLU	2013
1326009	SLU	2013
1337013	SLU	2013
1337015	SLU	2013
1337017	SLU	2013

1342004	SLU	2013
1402001	SLU	2014
1402003	SLU	2014
1402009	SLU	2014
1410004	SLU	2014
1410005	SLU	2014
1414011	SLU	2014
1415001	SLU	2014
1415003	SLU	2014
1419002	SLU	2014
1419004	SLU	2014
1419006	SLU	2014
1419009	SLU	2014
1419010	SLU	2014
1429006	SLU	2014
1433004	SLU	2014
1433005	SLU	2014
1433006	SLU	2014
1438004	SLU	2014
1442007	SLU	2014
1452001	SLU	2014
2-IV-4	SLU CWR	N/A
2-IV-6	SLU CWR	N/A
N5-18	SLU CWR	N/A
16	SLU*	
96	SLU*	
97	SLU*	

107	SLU*	
121	SLU*	
127	SLU*	
131	SLU*	
188	SLU*	
Blå Mandel	SWE	N/A
Blaue Schweden	SWE	1800
Mandel	SWE	N/A
Maria	SWE	1972
Perlo	SWE	2007
Röda krokar	SWE	1800
Sparris	SWE	N/A
Tärendö	SWE	N/A
Magda	TCH	2000
Early Puritan	USA	1988
Early Rose	USA	1897
Purple Majesty	USA	N/A

AUT = Austria, BRD = Germany, CH = Switzerland, DDR = former East Germany, DK = Denmark, FIN = Finland, FRA = France, GB = Great Britain, IRL = Ireland, IS = Iceland, NDL = The Netherlands, NOR/POL = Norway/Poland, SWE = Sweden, TCH = Czechia, USA = United States of America, N/A = unavailable, SLU CWR = SLU's crop wild relative-derived breeding clone, SLU* = SLU's chip potato breeding clone

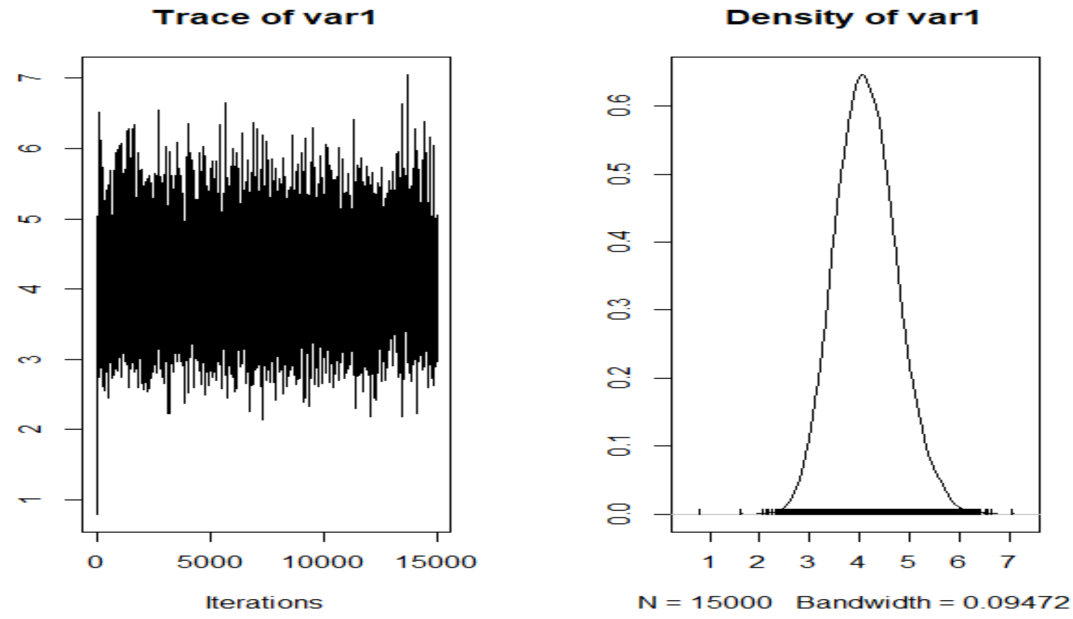

Figure S1. Markov Change Monte Carlo (MCMC) diagnostics. Trace of variance and density distribution indicates whether a large portion of the MCMC sample has been drawn from distributions that are significantly different from the target distribution.

Table S2. Phenotypic correlations between 3 sites for 6 traits (total tuber weight, and weight for tubers < 40 mm, 40–50 mm, 50–60 mm, > 60 mm, and starch).

Total tuber weigth

	Helgegården	Mosslunda	Umeå
Helgegården	1	0.6595865	0.69971255
Mosslunda	0.65958650	1	0.64876088
Umeå	0.69971255	0.64876088	1

Weigth for tubers < 40 mm

	Helgegården	Mosslunda	Umeå
Helgegården	1	0.59940731	0.68341362
Mosslunda	0.59940731	1	0.57811271
Umeå	0.68341362	0.57811271	1

Weigth for tubers 40–50 mm

	Helgegården	Mosslunda	Umeå
Helgegården	1	0.44996004	0.12189786
Mosslunda	0.44996004	1	0.37558829
Umeå	0.12189786	0.37558829	1

Weigth for tubers 50–60 mm

	Helgegården	Mosslunda	Umeå
Helgegården	1	0.53769307	0.44646189
Mosslunda	0.53769307	1	0.69092806
Umeå	0.44646189	0.69092806	1

Weigth for tubers > 60 mm

	Helgegården	Mosslunda	Umeå
Helgegården	1	0.69371655	0.69838046
Mosslunda	0.69371655	1	0.66104315
Umeå	0.69838046	0.66104315	1

Starch

	Helgegården	Mosslunda	Umeå
Helgegården	1	0.83608897	0.85104941
Mosslunda	0.83608897	1	0.77242877
Umeå	0.85104941	0.77242877	1
